# Advances in Research on Southern Corn Rust, a Devasting Fungal Disease

**DOI:** 10.3390/ijms252413644

**Published:** 2024-12-20

**Authors:** Yanyong Cao, Zeqiang Cheng, Juan Ma, Wenbo Yang, Xueman Liu, Xuan Zhang, Jinghua Zhang, Xiaolin Wu, Canxing Duan

**Affiliations:** 1 The Shennong Laboratory, Institute of Cereal Crops, Henan Academy of Agricultural Sciences, Zhengzhou 450002, China; yanyongcao@126.com (Y.C.); zeqiangcheng@163.com (Z.C.); majuanjuan85@126.com (J.M.); bbg.123@163.com (W.Y.); 2 National Key Laboratory of Wheat and Maize Crop Science, College of Life Sciences, Henan Agricultural University, Zhengzhou 450046, China; 13783990426@163.com (X.L.); zhangxuan0280@163.com (X.Z.); zhangjinghua2016@163.com (J.Z.); 3 Key Laboratory of Grain Crop Genetic Resources Evaluation and Utilization, Institute of Crop Sciences, Chinese Academy of Agricultural Sciences, Beijing 100081, China

**Keywords:** southern corn rust (SCR), *Puccinia polysora*, resistant germplasm resources, resistant genes/QTL, prevention and control strategies

## Abstract

Southern corn rust (SCR), caused by the obligate biotrophic fungus *Puccinia polysora* Underw., represents one of the most devastating threats to maize production, potentially resulting in yield losses exceeding 50%. Due to global climate change and cropping practices, epiphytotics of SCR have been increasingly reported, and are progressively spreading from tropical and subtropical maize growing areas to higher latitude areas. Over the past decade, researchers worldwide have undertaken extensive investigations into SCR, encompassing its occurrence and transmission pathways, the causative pathogen, the identification of resistant/tolerant germplasms along with associated genes/QTL, as well as potential control strategies. Nevertheless, information pertaining to this disease remains fragmented; thus far, standardized preventive and control measures have yet to be established. In response to this situation, this review seeks to comprehensively synthesize research findings on SCR while providing valuable insights into its occurrence, prevention, and control strategies aimed at mitigating the adverse impact and losses caused by SCR on global maize production.

## 1. Introduction

Maize (*Zea mays* L.) is one of the most widely cultivated crops globally, playing an increasingly diverse role in global agri-food systems and food/nutrition security [1,2]. However, maize production is threatened by various diseases, with one of the major challenges being southern corn rust (SCR), an airborne disease of maize caused by the obligate biotrophic fungus *Puccinia polysora* Underw. (*P. polysora*), which frequently occurs in tropical and subtropical maize growing areas. Under favorable conditions, although SCR can also occur in temperate regions, the pathogen typically cannot overwinter except in rare instances and must be reintroduced each growing season [3]. Owing to global climate change and the lack of SCR-resistant maize cultivars, epidemics of SCR have been reported more frequently, and are gradually spreading to higher latitude areas recently [4,5]. Up to now, SCR is prevalent in almost all maize-producing areas covering Africa, Asia, the Americas, and Australasia and has been reported to cause significant yield and economic losses [6,7]. For example, it is estimated that in Georgia, USA, SCR caused damages of more than 18 million dollars in 2014 [8]; also, in 2016, the maize yield loss attributed to SCR was estimated to be 20.783 million bushels in the southern United States, ranking as the second most serious disease in terms of yield loss [5]. Under optimal conditions, outbreaks and epidemics of SCR can result in yield losses exceeding 50% [9,10,11,12,13,14], with a higher loss occurring in late-planted maize, posing a serious threat to local maize cultivation and food security. Consequently, SCR has emerged as one of the most devastating diseases affecting maize across nearly all major producing areas globally [6,15,16,17]. Therefore, effective control measures against SCR are essential for sustainable global maize production.

Over the past decade, considerable efforts have been made to understand the occurrence of SCR, the causative pathogen (e.g., [18,19], the identification of resistant/tolerant germplasms along with associated genes/QTL (e.g., [20,21,22,23,24,25]), as well as potential control strategies (e.g., [26,27]. However, to date, there remains a lack of broad consensus on SCR and effective control measures due to multiple factors such as the rapid evolution of the pathogen along with its multiple physiological races, long-distance migration patterns, and the lack of varieties resistant to SCR [6,15,28,29,30,31,32], which may contribute to the ongoing increase in yield losses. Thus, to deepen our understanding of the pathogen responsible for SCR resistance, we present this review to attempt to comprehensively synthesize research findings on SCR. Additionally, we also outline effective disease management strategies to alleviate the adverse impact and losses caused by SCR on global maize production and propose a future outlook.

## 2. Occurrence and Threats of SCR

The SCR pathogen, *P. polysora*, was first identified and described in 1891 from a herbarium specimen of *T. dactyloides* in Alabama, USA and was formally named in 1897 [33]. However, its earlier presence has been confirmed through the examination of herbarium specimens dating back to 1879 in Central and Southern America [34]. The disease raised considerable alarm when it unexpectedly emerged in West Africa in 1949, where severe epidemics spread rapidly, resulting in significant yield losses in Sierra Leone and other countries [9], and quickly spread across the continent [35]. In 1953, SCR manifested violently at various widely separated locations within the Indian Ocean area, including Mauritius as the easternmost point of disease migration, which constituted a menace to maize production in Asia and Australia [36]. That same year, SCR outbreaks also occurred in the Philippines, where very large yield losses (80–84%) were found on susceptible cultivars [10]. Subsequently, SCR rapidly spread to India [37], Australia [38,39], Thailand [40], Japan [41,42], and China [43], among others. To date, SCR has disseminated across more than 110 countries spanning Africa, Asia, the Americas, and Australasia; it is now classified as a major global disease [6,7]. Furthermore, on account of rising global temperatures, SCR is projected to migrate poleward by an average of 15° by the year 2100 while exhibiting trends toward a general reduction in incidence within the Southern hemisphere alongside gradual increases within the Northern hemisphere [16].

In China, SCR was first identified in Hainan Province in 1972 [43]. Since 1998, outbreaks of the disease progressively moved northward, resulting in substantial yield losses across Hebei, Jiangsu, Zhejiang, Shanxi, Shandong, and Henan provinces, and so on [6,13,44,45]. To date, SCR has been widely distributed in China and covered more than 20 provinces/cities [6]. Factors such as climate change, changes in farming practices, and the cultivation of susceptible varieties over large areas exacerbate the risk of SCR outbreaks in China [6,44].

## 3. SCR Characterization

### 3.1. Pathogen

Since the emergence of SCR, it has posed significant threats to maize cultivation in tropical and subtropical regions. Its causal pathogen *P. polysora* is an obligate biotrophic parasite that infects, grows, and reproduces only on living maize plant tissues. Moreover, the pathogen also can infect *Euchlaena mexicana* (teosinte), *Erianthus divaricatus* (a species of grass related to the sugarcane), and four *Tripsacum* species (i.e., *T. laxum*, *T. latifolium*, *T. lanceolatum*, and *T. dactyloides*) [46]. *P. polysora* shows enormous destructive potential in maize. It infects all the aboveground parts of maize plants at any growth stage, especially young leaves, causing leaf necrosis and obstruction of photosynthesis until the plant dies [11,47]. Moreover, late-planted maize may be at greater risk for infection, and the disease attains its full severity at or about flowering time for the maize plant in most areas [11,12,48,49]. *P. polysora* produces fuzzy, raised structures called pustules primarily on the upper leaf surfaces and densely clustered, distinguishing them from *P. sorghi* causing common rust whose pustules develop on both the upper and lower leaf surfaces, tend to be spread out, and are not densely grouped [6,30,50,51]. In severe cases, *P. polysora* pustules may also develop on husks, leaf sheaths, and ear shanks [50]. Pustules (known as uredia) contain thousands of small orange to tan spores called urediniospores, which have the capacity for wind dispersal over long distances, extending to thousands of kilometers from the initial infection sites under high inoculum pressure [36].

*P. polysora* urediniospores are one-celled, light yellow to gold in color, ellipsoid, hyaline toward the outer member, and echinulate, with 4–5 equatorial pores [6,30,49,51]. Urediniospore germination, controlled by environmental temperature, relative humidity, and sunshine duration, is the initial step in infection to cause SCR [6,52,53,54]. Urediniospores can germinate in a range of 10–35 °C with the optimum temperature of 25 °C, while the germination percentage will be reduced greatly when below 15 °C or above 30 °C. The cardinal temperatures are 5 °C and 40 °C where urediniospores hardly germinated at different incubation times [54]. Yang et al. [55] also reported that the optimal temperatures for urediniospores germination and SCR development are 26–28 °C and 24–27 °C, respectively, and the temperature range for the disease occurrence and development is 15–31 °C. While *P. polysora* survives during humid seasons, the epidemic rate of SCR was negatively correlated with hours of relative humidity >90% [53]. Moreover, exposure of urediniospores to strong light could also inactivate rust fungi on plant surfaces or in the atmosphere [56]. It has been reported that the density of urediniospores of SCR in the air was significantly negatively correlated with temperatures above 27 °C and sunshine duration [52].

*P. polysora* can survive and repeat its asexual cycle and infection cycle through urediniospores throughout the year in tropical areas. In summer, urediniospores are dispersed by the wind currents from tropical areas to the north, where the pathogen cannot over-winter, and begin new infections [50]. When conditions are favorable, as short as nine days after the inoculation of a leaf surface with uredospores, new uredospores are produced and can be dispersed, which could cause devastating epidemics very quickly [46].

While late in the season, *P. polysora* may form brown to black pustules known as telia, which produces overwintering teliospores. Teliospores are two-celled, chestnut brown, angular to ellipsoid or oblong [6,49]. Teliospores are rarely or not produced at all in nature, are sometimes covered with an epidermis, and do not dehisce, which makes them difficult to detect [30,46]. Up to now, the germination of teliospores under experimental conditions has been unsuccessful [46]. Therefore, although teliospores may be of significance for assuring extended survival of the pathogen and developing new physiologic races [57,58,59], the function of teliospores in the life cycle remains speculative; experimental data on teliospore behavior should be critically reviewed.

Many rust fungi display complete a life cycle that involves five different spore types and stages: pycniospores (corresponding to pycnidial stage), aeciospores (aecidial stage), urediniospores (uredinial stage), teliospores (telial stage), and basidiospores (basidial stage), among which the aecia and pycnia stages occur on alternate hosts [60,61]. Among several cereal rust fungi, the host specificities and complete life cycles of *P. sorghi* causing common rust and *P. striiformis* causing wheat stripe rust are well understood [59,60,62]. However, the life cycle and mating system of *P. polysora* remain unknown to date, because of the absence of an alternate host and the aecidial and pycnidial stages [6,46,54]. Therefore, *P. polysora* was classified provisionally as a microcyclic and autoecious hamiform [46,63]. Recently, the reproduction modes of *P. polysora* in China were investigated based on genotype data, and the results showed that clonal reproduction of *P. polysora* was dominant [63,64]. Therefore, the reproductive modes of *P. polysora* populations require further investigation. A comprehensive understanding of the cycle and transmission mechanisms of SCR is essential for the efficient implementation of diagnostic and control strategies.

### 3.2. Genome and Effectors of P. polysora

Rust fungi are manifested by very large genomes (for example, from ~150 Mbp to over 1.0 Gbp) with large proportions of transposable elements and repetitive sequences, and two haploid nuclei in most life stages, which makes achieving high-quality genome assemblies challenging [65,66,67]. More recently, the genome of *P. polysora* was assembled by using HiFi reads and Hi-C data [68], and the results revealed that the final assembled genome is 1.71 Gbp, with ~850 Mbp and 18 chromosomes in each haplotype, currently one of the two giga-scale fungi assembled to chromosome level. The *P. polysora* genome displays over 85% repetitive sequences, which was mainly caused by Class I retrotransposons with long terminal repeats (LTR), with the LTR-Gypsy superfamily most abundant, and LTR-Copia next [68].

To infect plants, rust fungi secrete specific effectors, known as avirulence (*Avr*) genes, into host cells, which can be recognized by resistance proteins (R proteins) from resistant plants and trigger defense responses [69,70,71,72]. However, to escape this recognition and facilitate host plant susceptibility, phytopathogens have evolved diverse effector proteins and possess the ability to mutate *Avr* genes [18,73,74,75,76]. So far, only *AvrRppC* and *AvrRppK* effectors from *P. polysora* were cloned [18,19,71]. *AvrRppC*, anchoring on chromosome 14, encodes a 104 amino acid (aa) protein with a predicted signal peptide but no other known functional domains, nor any similarity to other known proteins [18,68]. AvrRppC interacts with RppC and induces RppC-dependent hypersensitive response (HR) in maize [18]. They are co-localized in the endoplasmic reticulum. *AvrRppC* has low expression in germinated spores and early infection but high expression in intermediate infection [68]. To date, 11 *AvrRppC* alleles have been found: *AvrRppC*^ref^, *AvrRppC*^A^, *AvrRppC*^C^, *AvrRppC*^E^, *AvrRppC*^F^, *AvrRppC*^J^, and *AvrRppC*^1^ to *AvrRppC*^5^ [18,68]. Allelic variation of *AvrRppC* directly determines the effectiveness of *RppC*-mediated resistance. Isolates with allele types of *AvrRppC*^ref^, *AvrRppC*^C^, and *AvrRppC*^E^ could activate *RppC*-mediated resistance, whereas *AvrRppC*^A^, *AvrRppC*^F^, and *AvrRppC*^J^ are virulent alleles that could escape the recognition of *RppC* causing SCR [18]. *AvrRppK*, encoding a 96 aa protein with a predicted signal peptide but also lacking any other known functional domains, is broadly distributed and highly conserved with no sequence variation in all examined *P. polysora* isolates. During infection, *AvrRppK* has a high express level and can suppress maize resistance against SCR and reduce chitin-triggered pattern-triggered immunity (PTI) [19], which is one of the two distinct immune reactions of the plant’s innate immunity and is triggered by the recognition of microbial components by cell surface-localized pattern-recognition receptors (PRRs), the other being effector-triggered immunity (ETI) activated by the recognition of microbial effector proteins directly or indirectly by intracellular nucleotide-binding leucine-rich repeat receptors (NLRs) [73]. Furthermore, with the sequencing of the *P. polysora* genome, candidate effectors can be predicted [68], which facilitates the identification of more *Avr* genes. In addition, Arndell et al. [77] developed a library screening platform to rapidly identify interacting pairs of plant immunoreceptors and pathogen Avr effectors that confer disease resistance, which may be applicable to the rapid identification of novel *Ave* genes from *P. polysora*.

### 3.3. Genetic Diversity of P. polysora

Studying the genetic population structure of *P. polysora* in a global context is important because the pathogen can easily spread from one country to another with wind currents and even inter-continental spread. However, information on the population structure and genetic diversity of *P. polysora* across large geographic distances remains limited.

Based on geographic distribution, more than 10 physiological races of *P. polysora* have been identified, including EA1, EA2, EA3, and PP.3–PP.9 [78,79,80,81,82]. Race EA1 was initially established from urediniospore collection samples in 1952 in Zanzibar, Tanganyika, and Uganda [78]. The EA2 race emerged spontaneously in the Muguga glasshouses in early 1955 [79], while the EA3 race was recognized in Kenya in 1961 [80]. In 1962, Robert discovered six new races, designated as PP. 3, PP. 4, PP. 5, PP. 6, PP. 7, and PP. 8 in North and Central America, with 11 maize cultivars as differentials [81]. The tenth race, PP.9, was identified by Ullstrup in South Africa in 1965 [82].

In Brazil, 17 virulence patterns were identified among 60 single pustule isolates of *P. polysora* from different areas [83]. In Japan, there exist at least 2 genetic groups based on genetic diversity analysis of 14 isolates using restricted fragment length polymorphisms (RFLP) of the internal transcribed spacer 1 (ITS1) and ITS2 regions [41]. In Thailand, based on the result of the genetic diversity of *P. polysora* using inter simple sequence repeat (ISSR) markers, 38 specimens from different provinces were divided into 13 groups, and the isolates from different localities were presented in the same groups, supporting the spore migration among different provinces in Thailand [40]. Genetic differentiation of *P. polysora* populations on 52 varieties of Kasetsart inbred lines (Ki1–Ki52) planted in different geographical localities of Thailand was analyzed using ISSR markers. The results showed that there was high genetic differentiation within *P. polysora* populations, but some genotypes were found in more than one province, indicating that the genetic diversity of the isolates has no correlation with their geographical distribution due to the migration of urediospores [84].

In China, the genetic diversity of 25 isolates of *P. polysora* collected in Hainan, Henan, and Chongqing provinces was analyzed using internal transcribed spacer (ITS) and *β-tubulin* sequences. Results revealed high similarities of 99.12–100% and 98.97–99.91%, respectively, indicating low genetic diversity for all isolates and a lack of regional differentiation [85]. Genome sequencing of 80 isolates expanded from south to north in China in recent decades also revealed low genetic differentiation in the Chinese *P. polysora* population. The majority of Chinese *P. polysora* populations carried the avirulence allele, *AvrRppC*^ref^, and only a minor population with low frequency (four isolates) is differentiated by carrying the virulent alleles, *AvrRppC*^A^ and *AvrRppC*^J^, indicating the ongoing virulence evolution to evade recognition by *RppC* [68]. However, in other studies, strong genotypic diversity was detected in China [63,64,86]. The genetic diversity of a total of 72 isolates collected from 12 provinces in China during 2011 and 2012 was studied using 18 polymorphic ISSR primers. The results showed that the 72 isolates could be clustered into 10 branches including 2 groups and 5 subgroups, with high genetic diversity, and that there exist some relationships in subgroups based on collected loci and years [86]. Recently, the population genetics of 96 isolates collected from the cities of Xinyi, Huizhou, Yangjiang, and Heyuan in Guangdong Province were investigated using 9 polymorphic simple sequence repeats (SSR) markers [63]. The results showed that the Yangjiang population had the highest genotypic diversity and the greatest number of multilocus genotypes, followed by the Heyuan, Huizhou, and Xinyi populations, and the isolates from different localities presented similar genetic characteristics, which might be due to spore migration. Using the nine SSR markers, Sun et al. [64] investigated the population structure and genetic diversity of 288 *P. polysora* isolates collected from various localities in 2017 in seven Chinese provinces: Guangxi, Guangdong, Anhui, Hunan, Shandong, Henan, and Shaanxi. The result displayed that strong genotypic diversity was detected, and the genetic diversity and genotypic richness of the populations showed significant spatial differentiation, with a decreasing trend from south to north, indicating that *P. polysora* employed clone dispersal from the pathogen’s winter reproductive regions to the pathogen’s epidemic regions in China. In addition, they also found that most isolates were clustered into two clonal groups, and two high-frequency multilocus genotypes (MLGs), MLG1 and MLG2, were widely distributed in all populations [64], suggesting *P. polysora* employed clone dispersal through wind in China.

Knowledge of the genetic diversity and genetic variation of *P. polysora* may provide clues about the prevalence and occurrence of SCR, which may contribute to more effective control measures. The genetic population structure and genetic diversity of *P. polysora* should be studied further.

### 3.4. P. polysora Infection Sources

Identifying the infection sources and dispersal pathways of *P. polysora* in the epidemic regions will provide an important theoretical reference for the formulation of disease management strategies.

*P. polysora* urediniospores can be dispersed by wind currents to altitudes exceeding 15,000 ft and distances of several hundreds of miles from their origin [36]. An examination of herbarium material of *P. polysora* from all areas of the world revealed two main size groups of urediniospores, a small size group in Southeast Asia and neighboring islands with the exception of Borneo, and a large size group in the West Indies, Africa, and the South Indian Ocean, which were consistent with the two directional spread, westwards and eastwards, from their origin in the Caribbean area [87]. However, as data on cross-inoculation studies are lacking, it is uncertain whether distinct forms of *P. polysora* exist. Cammack [88] inferred that urediniospores in the Caribbean were the sole source of infection dispersing to West Africa, as *P. polysora* was confined to the Caribbean area before 1949. According to Unartngam et al. [40] and Janruang et al. [84], *P. polysora* from different provinces in Thailand were divided into the same groups, supporting the long-distance migration of urediniospores. In Brazil, *P. polysora* from different areas had the same virulence phenotypes, suggesting the absence of geographical differentiation among prevalent populations of *P. polysora* in Brazil because of urediniospore migration [83].

In China, *P. polysora* can overwinter and survive throughout the year with a continuity of the uredinial stage in the southern areas, including Hainan, Guangdong, and Guangxi, where maize can be planted throughout the year; while for the major summer maize-producing area in the Huanghuaihai region where the pathogen cannot overwinter, SCR occurs only in summer [6]. However, the infection sources of *P. polysora* in China remain uncertain. *P. polysora* urediospores serve as both primary and secondary inoculum and are known to retain viability over long distances [15]. Tropical cyclones might carry the urediospores from southern China to northern China [89]. Liu et al. [44] inferred that the emergence of *P. polysora* in Tianjin was likely caused by the long-distance wind dispersal from southern China. Based on the study of the population genetics of *P. polysora* in China, Sun et al. [64] considered that *P. polysora* employed clone dispersal through wind from the pathogen’s winter-reproductive regions to the pathogen’s epidemic regions in China, since the isolates of *P. polysora* from the various provinces presented similar genetic characteristics and the genetic diversity and genotypic richness of the populations showed significant spatial differentiation, with a decreasing trend from south to north. Therefore, the pathogen in southern China may be the source of the disease occurring in summer maize in northern China [6].

However, some scholars conclude that the infection sources of the pathogen in mainland China are other areas outside mainland China [6,86,89,90]. Based on the result of the high genetic diversity of *P. polysora* among provincial populations, Guo et al. [86] inferred that the populations of *P. polysora* in Hainan, Guangdong, and Guangxi provinces were not the primary infection source in summer maize in the Huanghuaihai area, and the pathogen in China might originate from other areas outside the mainland of China. Similarly, using 11 ISSR markers to analyze the population genetics of 60 isolates, Yan et al. [90] found that the isolates collected in Shandong Province were highly similar to those collected from Jiangsu, Zhejiang, and Henan provinces, but the least similar to those collected in Hainan province. Therefore, they inferred that the primary infection sources of the disease occurring in Shandong province were probably the Philippines or Taiwan, China, rather than Hainan. Wang et al. [89] used 18 ISSR markers to study the population genetics of 75 isolates collected from different provinces and speculated that the infection of *P. polysora* in China is primarily multi-source. *P. polysora* comes from Taiwan, China, and the original source was found in the Huanghuaihai region and the Liaoning, Zhejiang, Fujian, and Guangdong provinces. The pathogen from the Philippines has its source in Guangdong, Guangxi, and Hainan provinces, and Thailand and other neighboring countries are the sources of the pathogen occurring in the Yunnan and Guizhou provinces [6,89]. More recently, according to Sun et al. [64], it was speculated that the infection sources in the pathogen’s epidemic regions did not all originate from the pathogen’s winter-reproductive regions, and the pathogen in Anhui and Hunan might also have other sources from areas such as Taiwan, China, and/or Southeast Asia since the diverse isolates sampled in July (69 isolates) were not grouped with those collected in April (200 isolates). However, there remains a lack of molecular evidence that the disease spread from Taiwan, China, the Philippines, and Thailand to mainland China, because these studies did not actually include any isolates from Taiwan, China, and Southeast Asian countries in their analyses for comparison [64]. Thus, it is necessary to collect more samples in China, especially in Taiwan, China, as well as in Southeast Asia to figure out the infection source and dispersal pathways of *P. polysora* in China.

## 4. Genetic Dissection of SCR Resistance

### 4.1. Resistant Maize Germplasm Resources

Adopting the cultivation of disease resistant maize varieties is considered an effective and environmentally friendly strategy for achieving durable control of SCR [71]. Thus far, the considerable efforts have mainly focused on identifying and screening SCR-resistant germplasm resources. Here, we systematically summarized the research progress in the identification of maize germplasm resources resistant to SCR.

In India, Kumar et al. [91] evaluated 60 indigenous and exotic maize inbred lines under artificial epiphytotic conditions and the results showed that 10 inbred lines were identified as resistant against SCR at Nagenahalli. Pati et al. [92] found that only genotype “71354” demonstrated the highest resistance after artificial inoculation of *P. polysora* among all the 15 maize genotypes screened in Manipur. In Brazil, Kurosawa et al. [93] evaluated 37 temperate and tropical popcorn genotypes under field conditions and natural inoculation in randomized block experiments with four replicates in two crop seasons in Campos dos Goytacazes, RJ, and found that the landraces PARA 172, ARZM 05083, and the line L80 are recommended for integration with breeding populations for SCR resistance as they may carry favorable alleles for disease control. In America, Chávez-Medina et al. [94] screened 1890 maize accessions for reactions to *P. polysora* race PP. 9 at Urbana and found 55 accessions rated 0.5 to 1 appeared to have some partial resistance to SCR.

In China, Chen et al. [95] evaluated the resistance of 10 maize inbred lines to *P. polysora* and found that Qi319 was highly resistant to SCR, followed by inbred line 178. Wang et al. [96] found that the resistant line Qi319 harbored the greatest number of resistance alleles (*n* = 70), followed by the CL11 line (*n* = 59) in 33 elite maize inbred lines, and recommended that the accumulation of additional resistance alleles in maize contributes to more robust SCR resistance. Wang et al. [97] investigated the resistance of 41 main cultivated maize varieties in China, and the result showed that only 4.88% (2/41) of varieties were highly resistant to SCR. Yuan et al. [98] found that Ludan981, Liyu16, and DH601 had a higher resistance to SCR in 19 maize varieties largely planted in Henan province and several main cultivated corn varieties, such as Zhengdan958, Ludan20, and Suyu2, were identified to be susceptible to SCR. Li and Du [99] screened 1218 maize germplasms using the artificial inoculation method, but only 24 genotypes (accounting for 1.97%) displayed a high level of resistance to SCR, and 39 genotypes (3.2%) exhibited resistant reactions. Chen et al. [100] evaluated 903 germplasm accession reactions to SCR in Nanning, Guangxi, and Changping, Beijing from 2013 to 2015, and the result showed that 8 inbred lines (accounting for 0.89%) were highly resistant and 29 inbred lines and landraces (3.21%) were resistant in the two independent replicated complete blocks. Huang [101] evaluated 1136 germplasm lines for SCR resistance using the artificial inoculation method from 2008 to 2010 and found that 28 germplasms (accounting for 2.46%) were highly resistant and 81 (7.13%) were resistant. Jiang et al. [102] screened 1589 maize germplasm accessions to SCR using Qi319 as the highly resistant control and Huangzaosi as the highly susceptible control by inoculation in the fields during 2008–2012 in Nanning, Guangxi. The results showed that 26 accessions were highly resistant, accounting for 1.64%, and 137 accessions were resistant, accounting for 8.62%. Du et al. [103] evaluated 379 germplasm lines for SCR resistance, containing 352 germplasm resources from the International Maize and Wheat Improvement Center (CIMMYT), 15 maize hybrids and 12 Chinese temperate maize inbred lines. The results displayed that 139 and 104 germplasms from CIMMYT were identified as highly resistant and resistant against SCR, respectively, just 2 Chinese temperate maize inbred lines were identified as highly resistant or resistant (one each), and 7 maize hybrids were resistant. Han [104] screened 103 maize inbred lines using the artificial inoculation method and identified 9 highly resistant inbred lines and 16 resistant germplasms. Yao et al. [105] identified 11 maize germplasms resistant to SCR, including 4 highly resistant germplasms, from 34 tropical or subtropical maize inbred lines. The four highly resistant germplasms were further studied through genetic analysis and the results indicated that there existed complex hereditary modes in the four germplasms, which were controlled by a host gene or a host gene combined with a minor gene. Mao et al. [106] evaluated 184 common and waxy maize inbred lines and found that 18 germplasms were highly resistant against *P. polysora* and 18 were resistant. Zhou et al. [107] evaluated the reactions of partial resistance to SCR in 253 maize inbred lines under natural infection and found that 18 lines (7.11%; 18/253) showed partial resistance to SCR, which was the same or significantly higher than the resistant control ‘Qi319’. Liu et al. [108] evaluated the resistance of 161 maize germplasms to *P. polysora* at the flowering stage in the field under natural infection and found that 69 germplasms were highly resistant and 31 were resistant. Meng and Huang [109] identified 5 inbred lines with high resistance to SCR in 76 exotic improved maize germplasms. Li et al. [110] evaluated 550 waxy maize germplasms and found that 2 germplasms were highly resistant against *P. polysora* and 40 were resistant. Tian et al. [111] tested 710 fresh maize inbred lines against SCR and found only 5 germplasms (0.7%; 5/710) were highly resistant under artificial inoculation and 30 (4.2%) germplasms were resistant.

In addition, some inbred lines such as AFRO.24 [78], W2D [112], CIMBL83 [113], J2416K [21], and Weiyu618 [114] were also found to be resistant germplasms. More details about the resistant sources are provided in Table 1.

These SCR-resistant germplasm resources can be used to identify resistant gene loci and cultivate resistant varieties that can effectively minimize the adverse effects of SCR on maize production at both molecular and population levels. However, in national and international studies on SCR disease, germplasm resources showing high resistance to SCR are scarce worldwide, especially in China. These Chinese SCR-resistant germplasm resources mainly come from the tropics and subtropics and are limited in a narrow genetic basis due to long-term breeding selection [115]. In addition, the resistance of maize cultivars is prone to loss due to the high genetic variability of *P. polysora* isolates. Thus, it is imperative to develop and identify the resistance of germplasm resources with diverse genetic backgrounds and mine durable resistance genes for disease-resistant breeding.

### 4.2. SCR-Resistance Genes/QTL

SCR resistance is a complex trait controlled by multiple genes [23], which necessitated the identification of quantitative trait loci (QTL) for initiating marker-assisted introgression of resistant QTL in elite susceptible inbred lines. In recent years, the identification of SCR resistance genes in maize has attracted considerable interest, including the disease resistance gene R, disease resistance regulatory genes, and QTL. So far, a number of genes/*QTL* responsible for SCR resistance have been mapped in resistant maize genotypes by using various methods.

On the basis of the race-specific resistant reactions of resistant maize genotypes against the 10 races of *P. polysora*, which contained 4 races EA1, EA2, EA3, and PP. 9 found in Africa [78,79,80,82] and 6 races PP. 3 to PP. 8 found in North and Central America [81], the resistance genes *Rpp1*–*Rpp11* were designated (Table 2). Among these, *Rpp1* and *Rpp2* were identified in the 1950s from maize lines AFRO.29 (‘Colombia 2’) and AFRO.24 (‘SLP 20-4A’) that originated in Colombia and Mexico, respectively, that conferred resistance in maize to races EA1 and EA2 [78]. *Rpp1* and *Rpp2* are linked and have an about 12% recombination frequency [116]. While both *Rpp1* and *Rpp2* were ineffective against race EA3 [80]. Genes *Rpp3* to *Rpp8* were identified by Robert [81] based on the differential response in 11 maize lines to 11 isolates of *P. polysora* collected from North and South America. *Rpp9*, a single dominant gene that conferred resistance to the race PP. 9, was identified by Ullstrup [82] from a South African maize cultivar PT186208. It is closely linked to *Rp1*, a maize gene for resistance to common maize rust on chromosome 10S [82]. The *Rpp9* gene has been used successfully in North America to control SCR for more than two decades [3]. However, due to the rapid evolution of the pathogen, *Rpp9* became ineffective in the southern US [15]. In addition, Futrell et al. [11] also report a single dominant gene for resistance to the race PP. 9 of *P. polysora* present in inbred line B1138TRpp that was derived from the Teko yellow open-pollinated cultivar, selected in Natal, South Africa, however, it was not tested whether the gene in B1138TRpp was allelic with the *Rpp9* gene. Genes *Rpp10* and *Rpp11* were recognized in maize lines AFRO.761 (*Andaqui*) and AFRO.600 (*Zapalote Chico*) that also originated in Colombia and Mexico, respectively [80]. *Rpp10* was fully dominant and afforded high resistance in maize to both EA1 and EA3, whereas *Rpp11* was incompletely dominant and conditioned an incomplete resistance to both races [80]. However, the genomic location of *Rpp1*–*Rpp11* had not been confirmed.

With the development of molecular markers, more and more resistance genes conferring resistance to SCR have been identified and fine-mapped by using different sets of maize germplasm (Table 2). Liu et al. [117] reported that resistance in the inbred line P25 was controlled by a major gene *RppP25*, which was roughly mapped on chromosome 10S with a genetic distance of 5.8 cM from the simple sequence repeat (SSR) marker phi059. In a subsequent study, *RppP25* was fine-mapped in the region between SSR markers P091 and M271, with an estimated length of 40 kb based on the physical map of B73 [22]. In this region, a candidate gene GRMZM2G060884 underlying *RppP25* was identified and is predicted to encode a putative nucleotide-binding site leucine-rich repeat (NBS-LRR) protein, a common characteristic of R genes. Chen et al. [118] indicated that resistance in the inbred line Qi319 was controlled by a dominant gene *RppQ*, which also maps on chromosome 10S between SSR marker phi041 and amplified fragment length polymorphism (AFLP) marker AF1 with a genetic distance of 2.45 and 3.34 cM, respectively. Subsequently, Zhou et al. [31] reported that the *RppQ* gene was located on the locus between sequence characterized amplified region (SCAR) marker MA7 and AFLP marker M-CCG/E-AGA_157_ with distances of 0.46 and 1.71 cM, respectively. *RppD*, a single dominant gene for resistance to SCR in the inbred line W2D, was also mapped on chromosome 10S in the region between SSR marker umc1291 and cleaved-amplified polymorphic sequence (CAPS) marker CAPS858, with genetic distances of 2.9 and 0.8 cM, respectively [112]. Moreover, through allelism tests, Zhang et al. [112] suggested that *RppD* might be a novel *Rpp* gene or haplotype differing from *RppQ* and *RppP25*. Yao et al. [119] indicated that resistance in the inbred line CML470 was controlled by the single dominant gene *RppCML470*, which was mapped on chromosome 10S in the region between SSR markers umc1380 and umc1291 with distances of 3.5 and 8.8 cM, respectively. *Rpp12*, a dominant gene for resistance to SCR in the inbred line Jiku12, was roughly located on the distal arm of chromosome 10S with 4.2 cM genetic distance from SSR marker phi063 [120]. In addition, through allelism tests, Zhang [120] suggested that *Rpp12* was non-allelic to the *RppQ* gene in Qi319. Wu et al. [121] reported that resistance against SCR in the tropical inbred line SCML205 was due to a single dominant gene *RppS*, which also maps on the distal arm of chromosome 10S 8.4 cM away from the marker IDP4283. More recently, according to Chen et al. [19], the *RppS* gene is an allele of *RppK* in K22, with only a 2bp-indel difference in the second intron region between the two alleles. *RppL2204*, a single dominant gene from Liao 2204, was located on the distal arm of chromosome 10S with a 9.6 cM genetic distance from SSR marker umc1380 [122]. Wang et al. [21] identified a single dominant gene, *RppM*, from the near-isogenic line Jing2416K that was immune to SCR. *RppM* was anchored to a 110 kb region between insertion/deletion (InDel) markers I15-5 and I16-4, in which two genes (*Zm00001d023265* and *Zm00001d023267*) encoding putative CC-NBS-LRR (coiled coiled, nucleotide-binding site, and leucine-rich repeat) proteins, a common characteristic of R genes, were identified. Subsequently, Wang et al. [123] further cloned *RppM* and found that it encoded a typical CC-NBS-LRR protein localized in both the nucleus and cytoplasm.

In recent years, QTL with partial resistance to SCR have been mapped to specific sites on chromosomes by linkage or association mapping using various mapping populations (Table 2). Jines et al. [124] identified 4 QTL with partial resistance to SCR, on chromosomes 4, 8, 9, and 10, respectively, by using recombinant inbred lines derived from a cross between a temperate-adapted all-tropical line NC300 and an Iowa StiV Stalk Synthetic line B104. A major QTL located on chromosome 10S, which explained 83% of the phenotypic variation, was positioned between markers UMC1380 and BNLG1451 (bins 10.0 and 10.1, respectively). Wanlayaporn et al. [24] reported 6 QTL on chromosomes 1, 2, 5, 6, 9, and 10 associated with partial resistance to SCR in a mapping population of tropical sweet corn recombinant inbred lines derived from a cross between hA9104 (resistant) and hA9035 (susceptible) inbred lines, and found that QTL on chromosome 1, 6, and 10 were stable QTL detected in different locations. By using a recombinant inbred population derived from the SCR-resistant line CML496 and susceptible line Lx9801, Lv et al. [125] mapped 3 QTL on chromosomes 6, 9, and 10, respectively. Among the three QTL, the major QTL *RppCML496,* located on chromosome 10, was consistently detected across environments, which accounted for 43–78% of the total phenotypic variation. *RppCML496* was further mapped to an interval of 128 Kb, in which a candidate gene *Zm00001d023311* with NBS-LRR domains was identified and considered to be the promising one for *RppCML496* against SCR. After further fine-mapping and genome sequencing of CML496, Deng et al. [18] narrowed the QTL region down to a 27.5 Kb interval, which contained only one NLR-encoding gene, *RppC*, and cloned it. RppC recognizes the avirulence effector AvrRppC secreted by *P. polysora* and mediates the resistance to SCR in maize. Chen et al. [19] identified one major QTL, *RppK*, on chromosome 10S that accounted for 68% of the phenotypic variation in resistance against SCR by using the F_6:7_ population generated by crossing K22 (SCR-resistant inbred line) with DAN340 (susceptible inbred line). *RppK* was further fine-mapped to an interval of ~18.3 Kb delimited by the markers SNP20 and SNP5 and cloned. It belongs to the CC-NLR gene family and can recognize the core effector AvrRppK of *P. polysora* to trigger ETI. Using recombinant inbred lines derived from a cross between susceptible inbred line Ye478 and resistant Qi319 in combination with their high-density genetic map, Lu et al. [23] located five QTL against SCR, designated as *qSCR3.04*, *qSCR5.07*, *qSCR6.01*, *qSCR9.03,* and *qSCR10.01*, on chromosomes 3, 5, 6, 9, and 10, respectively. Among the five QTL, the major QLT *qSCR6.01* detected on chromosome 6, with the highest effect value, accounting for 17.99–24.15% of total phenotypic variation in two environments, was linked to insertion-deletion markers Y6q77 and Y6q79, with physical locations of 77.6 and 79.6 Mb, respectively. Using the F_2_ population generated by crossing the highly resistant tropical maize line S313 with 4 highly susceptible inbred lines, Wang et al. [126] located a major QTL *RppS313* on chromosome 10S. *RppS313* was further mapped to a ~0.48 Mb region between SNP markers A005915 and A009920, in which three candidate genes LOC103640657, LOC100191493, and LOC103640673 encoding plant disease resistance-related proteins were predicted. Using the BC_2_F_5_ population derived from a cross between the resistant inbred line P178 with susceptible inbred line G41, Ai et al. [127] located five QTL against SCR, designated as *qSCR2.02/03*, *qSCR2.03/04*, *qSCR5.08*, *qSCR6.05,* and *qSCR10.01*, on chromosomes 2, 2, 5, 6, and 10, respectively, which explained 6.88–45.31% of total phenotypic variation. Among the five QTL, the major QLT *qSCR10.01* detected on chromosome 10S, with the highest effect value, was further mapped to a 1.34 Mb region between the markers UMC1380 and C(10)3595071. Using the F_2_ population derived from a cross between the highly resistant inbred line W456 with susceptible line Huangzaosi, Chen et al. [128] located six QTL against SCR, designated as *qSCR3* (between markers umc2105 and umc1729), *qSCR7* (between markers umc1066 and bnlg2271), *qSCR8-1* (between markers umc1904 and umc1984), *qSCR8-2* (between markers umc1984 and bnlg1651), *qSCR9* (between markers umc1957 and bnlg1401), and *qSCR10* (between markers umc2034 and umc1291), on chromosomes 3, 7, 8, 8, 9, and 10, respectively, which explained ~62.3% of total phenotypic variation. Among the six QTL, *qSCR10* on chromosome 10 accounted for 24.19% of the phenotypic variation, as a major QTL responsible for resistance to SCR, which was linked to markers umc2034 and umc1291 with genetic distances of 2.15 and 0.36 cM, respectively. Using BC_1_F_4_ population derived from a cross between the highly resistant inbred line TY4 with susceptible line Lx9801, Li et al. [129] detected six QTL against SCR, designated as *qSCR1.09*, *qSCR2.05*, *qSCR3.04*, *qSCR4.05*, *qSCR6.01,* and *qSCR9.03* on chromosomes 1, 2, 3, 4, 6, and 9, respectively, which explained 3.93–17.87% of total phenotypic variation. Among the six QTL, the stable QTL *qSCR6.01* (also named *RppT*) detected on chromosome 6, with the highest effect value, was finally narrowed down to an interval of 4.09 Mb delimited by the markers M3 and M4. Liu et al. [130] mapped two novel QTL, *qSCR4.05* and *qSCR4.08*, on chromosome 4, associated with resistance to SCR, via QTL mapping and Bulked Segregant RNA-Seq (BSR-Seq). Using a recombinant inbred line population derived from a cross between 975-12 (resistant) and Lx9801 (susceptible), four QTL were identified, which were located on chromosomes 5, 9, 9, and 10, respectively. Among them, a major QTL, *qSCR3*, on chromosome 10S, with the highest effect value, accounting for 70.3–78.4% of total phenotypic variation in three environments, was fine-mapped to an interval of ~225 Kb delimited by the markers MR10-2 and MR10-3, in which *Zm00025ab420970* encoded typical NLR proteins, was identified as the candidate gene for *qSCR3* by using transcripts assembly of 975-12 and its susceptible mutant 975-12S [131]. Using a recombinant inbred line population derived from a cross between CIMBL83 (resistant) and Lx9801 (susceptible), a major QTL, *qSCR4.01*, on chromosome 4, accounting for 70.3–78.4% of total phenotypic variation, was consistently detected across multiple environments. *qSCR4.01*, was delimited to an interval of ~770 Kb with flanking markers SOURST-83_2035716 and PZE-104005694 [113]. Based on bulked-segregant and transcriptome analysis, Mu et al. [132] identified eight QTL on chromosomes 1, 6, 8, and 10 contributing to SCR resistance using BC_4_F_1_ and BC_4_F_2_ populations derived from a cross between the resistant inbred line L119A with susceptible inbred line Lx9801, and observed extensive gene expression change in L119A but not in Lx9801. With the upregulation of lignin biosynthetic genes, as well as the specifically induced accumulation of lignin in L119A, Mu et al. [132] suggested that lignin plays a vital role in SCR resistance. By using a large F_2_ recombinant population derived from a cross between a maize landrace with high resistance to SCR ‘Silunuo’ (SLN) and a susceptible inbred line N531, Wang et al. [133] identified a major SCR-resistant locus, *RppSLN*, on chromosome 10, which accounted for 84.77% of the total phenotypic variation. *RppSLN* was ultimately narrowed down to an interval of 38 kb flanked by the markers W4 and W6, within which two NBS-LRR genes (*Zmays10G000430* and *Zmays10G000440*) were identified as the candidate genes. In addition, through map-based cloning, Wang et al. [134] cloned a teosinte-derived allele of a resistance gene, *Mexi cana lesion mimic 1* (*ZmMM1*), localized on bin 7.00, which causes alesion mimic phenotype and confers resistance to SCR, northern leaf blight (NLB), and gray leaf spot (GLS) in maize. *ZmMM1* encodes a transcription factor that contains a MYB DNA binding domain and negatively regulates the transcription of *ZmMM1-target gene 3* (*ZmMT3*), which encodes a lncRNA and negatively regulates such plant immune responses as PAMP-induced ROS accumulation.

Genome-wide association studies (GWAS) have also been successfully utilized to identify numerous candidate loci/genes associated with partial resistance to SCR (Table 3). Zhou et al. [107] performed a GWAS using the SNP3K beadchip and identified seven single nucleotide polymorphisms (SNPs) against SCR, on chromosomes 4, 8, and 10, from two environments on a panel of 253 maize inbred lines. de Souza Camacho et al. [135] performed a GWAS on a panel of 164 tropical inbred lines of maize and identified eight significant SNPs conferring SCR resistance on chromosomes 4, 5, 6, 7, 8, 9, and 10. Shu et al. [136] identified 13 QTNs for SCR resistance on chromosomes 1, 2, 4, 5, 6, and 8 based on multi-locus GWAS from a diversity panel of 140 inbred maize lines that include most of the parental lines of major commercial hybrids in the China Summer Corn Belt. Li et al. [137] identified five QTL on chromosomes 1, 7, 8, 8, and 10, using GWAS with a Doubled Haploid (DH) panel with 384 lines and a hybrid panel with 903 testcross hybrids. Among them, a highly significant locus on chromosome 10 was tight-chained with the known SCR resistance genes *RPPC* and *RPPK*. Oo et al. [138] performed a GWAS on a panel of 262 maize inbred lines and identified 19 SNPs/QTL distributed on chromosomes 1, 2, 3, 4, 5, 9, and 10 significantly associated with resistance to SCR disease. Sun et al. [32] conducted a GWAS on a panel of 752 temperate maize genotypes and identified four significant loci conferring SCR resistance on chromosomes 2, 4, 4, and 6. Wang et al. [96] performed a GWAS on a panel of 487 inbred maize lines cultivated in three distinct conditions and identified 91 loci substantially correlated with SCR susceptibility, among which 13 loci were significant in at least three environments, overlapping with 74 candidate genes.

Furthermore, there are also reports indicating the application of high-throughput sequencing in conjunction with bioinformatics analysis to investigate candidate genes associated with SCR resistance and the elucidation of the corresponding molecular mechanism. A comparative proteomic analysis of SCR-infected P178 (resistant line) and Lx9801 (susceptible line) plants revealed that the abundance of one remorin protein, ZmREM1.3, significantly increased in the SCR-resistant genotype, but decreased in the susceptible genotype [139]. Remorin gene *ZmREM1.3* overexpressing plants exhibited enhanced resistance against SCR and accumulated more salicylic acid (SA) and jasmonic acid (JA). *ZmREM1.3* positively regulates maize SCR resistance, likely via SA/JA-mediated defense signaling defense pathways. By integrating GWAS and transcriptomic datasets, Wang et al. [96] revealed *ZmHCT9*, encoding the protein hydroxycinnamoyl transferase 9, as the specific locus located on chromosome 1 that is linked to the negative regulation of SCR resistance in maize. In addition to conventional GWAS, Sun et al. [32] also performed a transcriptome-wide association study to identify additional genes associated with SCR disease severity and found the five most significantly associated genes, *Zm00001d035185*, *Zm00001d050928*, *Zm00001d035195*, *Zm00001d039975*, and *Zm00001d013049*. In conjunction with QTL analysis, Wang et al. [133] further conducted a comparison dynamic transcriptome analysis between a pair of near-isogenic lines, N531_R (highly resistant to SCR) and N531_S (highly susceptible to SCR) following *P. polysora* infection and identified 11 related genes exhibiting similar expression patterns to that of the key candidate gene *Zmays10G000440* of *RppSLN*, among which *Zmays01G042730* (similar to *AtPRL1*, a WD40-containing protein) and *Zmays01G009610* (identified as DNAJ heat shock protein 40) are directly related to the disease resistance effect. More recently, Yan et al. [140] dissected the heterogeneity of maize’s response to *P. polysora* infection using single-cell RNA sequencing and successfully identified resistance genes to SCR. The candidate gene *ZmCHit7*, a glycoside hydrolase family 18 chitinase 7 protein, was highly upregulated in multiple cell types under SCR infection. Overexpressing of *ZmCHit7* enhanced resistance against SCR, while the knockout lines were more sensitive to SCR. Furthermore, RNA-seq was performed in two *ZmChit7*-R99 overexpression lines and it was found that *ZmChit7*-R99 overexpression activated pathways like plant hormone signaling, plant–pathogen interaction, and MAPK signaling, suggesting that it positively regulates maize resistance to SCR, likely through a hormone-mediated defense pathway. Overall, omics data provide potential gene targets for manipulating SCR resistance in maize.

In light of these studies, while R genes or QTL have been identified and mapped on all maize chromosomes, chromosome 10 contains the largest number of R genes or major QTL, and molecular genetic analyses have revealed that a significant proportion of the identified/cloned R genes or major QTL are localized on chromosome 10S in a cluster consisting of allelic or tightly linked genes [115,125]. Due to the rapid evolution of the pathogen of SCR along with its multiple physiological races, relying on a single major QTL/gene could carry the risk of resistance breakdown. Therefore, further studies on discovering new sources associated with SCR resistance are still underway. Additionally, QTL data from different studies can be further integrated by meta-QTL analysis to mine the candidate genes and develop a constitutive gene network for disease resistance in maize [141]. There has been no report so far on the successful application of Meta-QTL in SCR resistance research. Nonetheless, this method holds great potential to provide novel insights into the genetic and molecular mechanisms of SCR resistance in maize for a more targeted improvement of this important trait in breeding. In brief, the identification and cloning of SCR resistance loci provide significant guidance for marker-assisted selection breeding to develop maize varieties that maintain productivity even under disease pressure.

## 5. Prevention and Control Strategies

Due to its devastating nature and rapid spread, SCR is a great threat, not only to food security but also to livelihoods worldwide. To control its spread and reduce SCR severity to some extent, a combination of multiple strategies is crucial.

It is well known that the most economically viable and environmentally sustainable approach for the control of SCR is to deploy SCR-resistant cultivars and hybrids. Over the years, by using the limited SCR-resistant maize germplasm resources, some elite maize inbred lines have been bred and successfully used for the breeding of SCR-resistant hybrids [115]. For instance, by using the resistant inbred line K22, carrying *RppK*, derived from a K11 × Ye478 cross, Chen et al. [19] performed repeated backcrossing and molecular marker-assisted selection to introduce the *RppK* gene into the two parental lines of the maize hybrid JK968, which has been planted several million hectares in China over the past decade [142]. And the result showed that the JK968 hybrid lines carrying *RppK* were more resistant to SCR and the grain yields of the JK968 lines carrying *RppK* were increased by more than 10% in the presence of SCR. Zhang et al. [143] introgressed the SCR-resistant gene *RppQ* from Qi319 into four elite sweet corn inbred lines using marker-assisted backcross breeding, and the converted lines and derived hybrids showed significantly enhanced resistance to SCR. The utilization of molecular marker-assisted selection in indirect breeding can significantly enhance the productivity and precision of conventional plant breeding programs [115,144]. Consequently, the integration of molecular marker-assisted breeding into traditional breeding programs represents a more effective strategy for improving maize resistance against SCR. Furthermore, CRISPR/Cas genome editing technologies, known for their flexibility, high efficiency, and versatility, have been proven to play a crucial role in developing disease resistance against bacteria, fungi, viruses, and other pathogens [145]. The molecular identification of maize genes that are essential for SCR infection but dispensable for plant growth and development is of significant interest in the development of CRISPR/ Cas-mediated resistance in maize. There are very few cases reported so far on the successful application of genome-editing technology in SCR research. Nonetheless, this method has great potential in breeding novel maize varieties with enhanced SCR resistance.

Effective field management practices, such as implementing suitable crop rotation and/or intercropping strategies, along with optimizing planting density, can mitigate the severity of SCR [6,28,146].

The application of chemical fungicides has a significant impact on the incidence and severity of SCR [147]. During the flare opening stage, the use of 40% syringandrol-tebuconazole with 0.01% brassinolide or 25% pyrazoxystrobin with 0.01% brassinolide can effectively control the outbreak of SCR [148]. Zhao et al. [149] suggested that using 75% pyrazole·azoxystrobin at the tasseling stage can effectively reduce SCR. A study has shown that the use of 30% trifloxystrobin·tebuconazole SC can effectively reduce SCR symptoms in fresh corn by up to 93% [150]. Moratelli et al. [26] found the mixture of triazole with estrobirulina is feasible to control the severity of SCR in maize. Faske and Emerson [151] suggested that using quinone outside inhibitor (QoI) or QoI + demethylation inhibitor fungicides at the tasseling stage effectively controlled SCR damage when SCR was present and environmental conditions favored rust development. Chemical control of SCR is regarded as the most effective solution in highly infected areas to protect susceptible maize cultivars. However, intensive application of chemical fungicides may cause the appearance of fungicide resistance and lead to human, animal, and environmental risks in the long term.

Biological control is considered a promising alternative and eco-friendly approach for plant disease management by inhibiting plant pathogens and/or improving plant immunity [147,152]. Zhao et al. [153] reported that the application of biocontrol strain R4 can inhibit SCR and increase maize yields. Silva et al. [154] reported that *Doru luteipes* fungivory may play an important role in the biological control of SCR. However, to enable this method of implementation, many knowledge gaps need to be addressed.

Disease forecast and SCR monitoring in field conditions could assist in making smart agriculture decisions and reducing pesticide inputs [17,155]. Meng et al. [27] developed spectral disease indices (SDIs)-based monitoring models to detect SCR-infected leaves and classify SCR damage severity, which provided a theoretical basis for remote sensing of SCR in the field over large areas. Lv et al. [155] introduced a novel method combining unmanned aerial vehicle (UAV)-based imaging spectral measurements with an attention-based Fully Connected Network (FCN) to improve monitoring of SCR, considering the bottom-up pathogenesis of SCR and canopy occlusion in the field. Additionally, Li et al. [52] established a prediction mode for SCR prediction based on the correlation between *P. polysora* spore number and disease index and meteorological factors. Yang et al. [156] developed an ecoclimatic index risk prediction (EIRP) model for the SCR that effectively simulates the biological mechanisms underlying infection dynamics, demonstrating high sensitivity and accuracy in predicting the distribution range, adaptability, and disease index of SCR in northern China.

## 6. Conclusions and Future Perspectives

SCR is a devastating disease that incurs significant economic costs in infected areas. In this review, we summarize the current understanding of SCR occurrence, the causative pathogen, the identification of resistant/tolerant germplasms and associated genes/QTL, as well as strategies for monitoring and mitigating its spread. Nevertheless, SCR continues to pose a significant threat to maize production, and the molecular and genetic mechanisms underlying resistance to SCR remain inadequately explored. The primary method for controlling SCR, the utilization of resistant germplasm, represents an effective, cost-efficient, and environmentally sustainable solution that requires ongoing enhancement and preservation efforts. However, there is limited information regarding the inheritance patterns of SCR resistance. Sources of maize genetic resistance must be pursued, more resistant genes/QTL should be identified and cloned, resistance mechanisms should be explored and clarified, and methods for rapidly identifying new stable cultivars with SCR resistance need development or improvement, novel breeding strategies for improving maize resistance against SCR should be developed and applicated. Alongside the primary approach, there is an urgent need for continued development of alternative solutions to effectively control the SCR pathogen. For instance, further identification and development of biopesticides against SCR are necessary. Biological control is receiving increasing attention as an alternative means of disease control; however, it is generally still in the experimental stage, and few biological control agents are currently available for the maize production system. Comprehensive research on the pathogenicity mechanism of *P. polysora* at a molecular level should be carried out, including plant–pathogen interactions, their mechanisms, and evolutionary adaptations. Moreover, methods or equipment for predicting and monitoring SCR under field conditions must be developed or improved upon. Thus, further efforts are required to gain deeper insights into SCR and achieve effective disease management.

## Figures and Tables

**Table 1:** Resistant sources to SCR reported across different countries.

Country	Resistant Material		References
	High Resistance	Resistance	
India	71354	Not mentioned	[92]
India	Not mentioned	NAI-104, 116, 117, 124, 137, 148, CM-212, CM-501, V-336 V-341	[91]
Brazil	L80, ARZM05083, PARA172	L63, L65, L76, P1, P4, ARZM07049, URUG 298 AMARELO, URUG 298 ROXO	[93]
America	Va59, IA DS61, Yellow Flint, Inbred 2-687, 4F-374 GE 3, Inbred 627, Puerto Rico 13, Loreto 9, Loreto 11, San Martin 126, OC5, Mo6, OC15, Kyles Long Ear, Va21A, Va22B, C103, Guerrero 3, INB 101LFY, Maiz Amarillo Klein, Mkt. Asuncion, Niksar Tokat, Arnet Damascus, Inbred 378, Inbred 334, No. 8815, No. 9179, No. 9180, Various markets, 4F-35 BK, 4F-240 BX 16, No. 162, No. 1122, No. 1126, Ladyfinger, E292, A256-1, E680, No. 9, Pukanki Zolti 1294, Antioquia 308, Antioquia 373, Antioquia 377, Atlatnico 308, Bolivar 308, Bolivar 326, Huil 1351, Magdelena 310, Magdelena 362, Tolima 403, Valle 343, Comum 094-R2, Lima 86, Loreto 8, San Martin 7, San Martin 9, Mo17, San Martin 111, Narino 628, BOZM 1155, Ky228	Not mentioned	[94]
China	Qi319	178	[95]
China	Qi319	CL11	[96]
China	Ludan981, Ludan50	Yuyu22, Huidan4, Hudan2000	[97]
China	Ludan981, Liyu16, DH601		[98]
China	Qi319, X178, N223, CA091, Liao2202, Qi318, Shuang M9B-1, Zong548-1521, L005, K36, Liao2204, Zun90110, Huapei09 up A196, Huapei09 up A242, 85Bai16, LO932, A31, A67, A69, A82, A101, A110, A210, A224	N321, N324, N338, JiTian15, Liao4271, Liao7794, ZhongXi042, Ji880, Fu8538, Lu65, Zhengbai11, FR218, M256, Huapei09 up A818, CS801, XS801, Ludan981, Nongda108, Huidan4, 4026, A16, A25, A52, A59, A73, A104, B781, Weichun3, A145, A156, A164, A182, A203, Bianyao13, A225, A252, Paobaogu, Huangbaogu, ChiL028	[99]
China	Ji186, 664062, Shen11-17, 95036, 164, 3271, 5304-48, Dan3130, 65 Chang35, Liao2202, Liao2204, YH09-272, Zun90110, A69, A101, A104, 5363, 5364, K36, P25, ShuangM9B-1, XiaoBaiZi, BaShiTian, BaiYuMi, XiaoHuangYuMi, JinHuang55, W456, SW15, SW19, SW21, LO932, SW94, SW107, SW113, SW114, SW115, CA091, CML180, Chi556	JiKu6, JiKu12, 5362, 5041, 5042, ChiShuiBai, BaiBaoGu, Lu9801, ZiBaoGu, JinHuangZao, entry02, X178, KH13, CI24, Liao5088, Liao51, ChiL382, CT3354, D9C2, 20104046, 9872, 12084, 6969, ChiH16, ChiH7, I62, Chi005, Chi007, Dan 79-1, BaXi 501, Chi74521, ReDai-1, 08F65, 9d1, S7, Bai p, 03Chang130, 05-949	[100]
China	A69, A82, A101, A104, Baiyumi, Bashitian, Xiaobaizi, Xiaohuangyumi, Szylaecka, Liao2202, Qi318, ShuangM9B-1, K36, Liao2204, Zun90110, 178, 5362, 5363, 5364, 5304-48, W456, SW-94, SW-113, SW-115, 2202, CA091, LO932		[101]
China	X178, K36, Zun90110, W456, Liao2202, Liao2204, ShuangM9B-1, Chi556, Chi547, Liao2201, SW-40, SW-94, SW-107, SW-113, SW-115, 3271, NX3, 70391, Laolaibi, Bashitian, Xiaobaizi, A69, A82, A101, A104, Dr11		[102]
China	139 germplasms from CIMMYT (such as CML161, CML204, CML286, W-98, SW-113, CI181); P138 (Chinese temperate maize inbred line)	104 germplasms from CIMMYT (such as CML165, CML268, CML285, CI197); 7 maize hybrids (i.e., Chenyu201, Chengdan22, Dika008, Guidan0810, Nongda108, Yumeitou105, Zhengda619); X178 (Chinese temperate maize inbred line)	[103]
China	CML305, CML307, CML411, CML470, CML496, CML497, S37, P178, K22	526018, P138, Dan360, B77, Ye478, Yu87-1, 7884-4Ht, CA47, CML115, CML360, Dan599, Zhong69, ZZ01, A619, D863F, GEMS41	[104]
China	CML144, CML247, CML451, CML470	CML159, CML161, CML206, CML387, CML395, CML491, CML496	[105]
China	K22, Qi319, ZGF, JHF, T178, R3, R4, R5, R7, R9, R11, N1, N24, Z25M, T43.7, T458, T75, T2	Nongda1145, Zhong128, DH02, 9409F, 4377, Z25F, H04-24, T1013, T1016, R2, R6, R10, N11, N23, N33, R-8, N1009, N1012	[106]
China	43.7, DH02, Zheng39, T2, JH3372, K22, P138, S2, ZGF, CM, T178, Zhong128, JS06730, N1, Qi319·X7, N24, S6, T75	Not mentioned	[107]
China	Bao335, M36, Hua168-1, Dongzheng1, Tai99-2	3104, Tai3-1, M8111, TS3926, TS771	[109]
China	KF6717, 494-2	40 germplasms (such as P19-713-10, TZihongnuo)	[110]
China	Waxy corn: 11N7-1-1, ZQN9-1-1-1, 09N1-1-1; Sweet corn: Z01-1-1; Sweet-waxy corn: ZTN9-1-1-2	Waxy corn: ZTN-1-1, 12N3-2, 09N17-1-1, JN-1-1-2, HHN09-1-1-1, KN45-2-1, KN7-1-1, KN-1-1-3, DN-8-1-1, KN56-1-1, FN9-1-1, FN8-1-2-1, FN2-1-2, ZQN8-1-1-1; Sweet corn: 10T5-1-2-1, QT7-1-2-2, WST-1-1, Z95, XZT-1-1-1, 11T8-1-1-1, KT38-1-1-1, 09T15-1-2-1, Z145-1-1-2; Sweet-waxy corn: S20, TN14-2-2-2, S15, TN6-2-1-2, TN12-2-1-2, TN27-3-1, TN72-2-1	[111]

**Table 2:** Summary of major genes/QTL associated with SCR resistance in maize.

SCR-Resistant Lines	Genes/QTL	Chr. ^a^	Location	PVE ^b^	References
AFRO.29	*Rpp1*				[78,116]
AFRO.24	*Rpp2*				[78,116]
	*Rpp3–Rpp8*				[81]
PT186208	*Rpp9*	10S			[11]
AFRO.761	*Rpp10*				[80]
AFRO.600	*Rpp11*				[80]
P25	*RppP25*	10S	Between SSR markers P091 and M271, with an estimated length of 40 kb		[22,117]
Qi319	*RppQ*	10S	Between SCAR marker MA7 and AFLP marker M-CCG/E-AGA157 with distances of 0.46 and 1.71 cM, respectively		[31,118]
W2D	*RppD*	10S	Between SSR marker umc1291 and CAPS marker CAPS858, with genetic distances of 2.9 and 0.8 cM, respectively		[112]
CML470	*RppCML470*	10S	Between SSR marker umc1380 and umc1291 with distances of 3.5 and 8.8 cM, respectively		[119]
Jiku12	*Rpp12*	10S	On the distal arm of chromosome 10 with 4.2 cM genetic distance from SSR marker phi063		[120]
SCML205	*RppS* (an allele of *RppK*)	10S	On the distal arm of chromosome 10S with 8.4 cM away from the marker IDP4283		[19,121]
Liao 2204	*RppL2204*	10S	On the distal arm of chromosome 10 with 9.6 cM genetic distance from SSR marker umc1380		[122]
Jing2416K	*RppM* *	10S	Anchored to a 110 kb region between InDel markers I15-5 and I16-4		[21,123]
NC300		10S	Between markers UMC1380 and BNLG1451 (bins 10.0 and 10.1, respectively)	83%	[124]
hA9104		1	Between markers umc2025 and umc1919	17.6–22.1%	[24]
		6	Between markers umc1614-umc1250	7.0–7.4%	
		10	Between markers umc1246-umc1239	15.1–22.0%	
CML496	*RppC* * (also named as *RppCML496*)	10	Mapped to an interval of 27.5 Kb between markers SSR-C2 and CRS-2839039	43–78%	[18,125]
K22	*RppK* *	10S	Mapped to an interval of ~18.3 Kb delimited by the markers SNP20 and SNP5	68%	[19]
Qi319	*qSCR6.01*	6	Between markers Y6q77 and Y6q79, with physical locations of 77.6 and 79.6 Mb, respectively	17.99–24.15%	[23]
S313	*RppS313*	10S	Mapped to a ~0.48 Mb region between SNP markers A005915 and A009920	83.1%	[126]
P178	*qSCR10.01*	10S	Mapped to a 1.34 Mb region between the markers UMC1380 and C(10)3595071	45.31%	[127]
W456	*qSCR10*	10	Between markers umc2034 and umc1291, with genetic distances of 2.15 and 0.36 cM, respectively	24.19%	[128]
TY4	*qSCR6.01* (also named as *RppT*)	6	Mapped to an interval of 4. 09 Mb delimited by the markers M3 and M4	17.87%	[129]
CT3354	*qSCR4.05*	4	Between markers AX-86269884 and AX-108026358	18.3%	[130]
	*qSCR4.08*	4	Between markers X-91851815 and AX-107983145	11.2%	
975-12	*qSCR3*	10	Mapped to a ~225 Kb region flanked by MR10–2 and MR10-3	70.3–78.4%	[131]
CIMBL83	*qSCR4.01*	4	Mapped to an interval of ~770 Kb with flanking markers SOURST-83_2035716 and PZE-104005694	48–65%	[113]
L119A	*QTL8*	10	Mapped to a 400 kb region (chromosome 8: 1,397,359–1,797,359)		[132]
Silunuo (SLN)	*RppSLN*	10	Mapped to an interval of 38 Kb with flanking markers W4 and W6	84.77%	[133]

^a^ Chr. = chromosomes; ^b^ PVE = Phenotypic variance of a single QTL; * represents the genes have been cloned.

**Table 3:** Summary of genetic loci associated with partial resistance to SCR identified by GWAS.

Test Materials	Loci Names (QTL or SNPs)	Chr. ^a^	Positions	Candidate Genes	References
253 maize inbred lines	*PZE-104026873*	4	Bin 4.04 (31,713,714)		[107]
	*PZE-108082079*	8	Bin 8.05 (138,887,412)		
	*PZE-108107270*	8	Bin 8.06 (161,428,052)		
	*PZE-108111762*	8	Bin 8.06 (164,040,056)		
	*SYN12403*	10	Bin 10.00 (2,658,622)		
	*SYN17109*	10	Bin 10.01 (3,989,555)		
	*PZE-110040601*	10	Bin 10.02 (77,534,916)		
164 tropical maize inbred lines	*S1_54225397*	10	Bin 10.03 (46,253,522)	*GRMZM2G015599*	[135]
	*S1_828487200*	7	Bin 7.05 (169,059,365)	*GRMZM2G451097*	
	*S1_195983129*	9	Bin 9.03 (38,378,950)	*GRMZM2G099745*	
	*S1_1010317140*	5	Bin 5.05 (174,062,894)	*GRMZM2G460958*	
	*S1_838749354*	5	Bin 5.00 (2,495,108)	*GRMZM2G119186*	
	*S1_574229676*	8	Bin 8.03 (90,179,433)	*GRMZM2G170167*	
	*S1_461974523*	6	Bin 6.05 (147,332,216)	*GRMZM2G133082*	
	*S1_1762313727*	4	Bin 4.10 (237,936,661)	*GRMZM5G851807*	
140 inbred maize lines	*S1_218*	1	218619398	*Zm00001d032240, Zm00001d032244*	[136]
	*S1_299b*	1	299623487	*Zm00001d034678*	
	*S2_12*	2	12916090	*Zm00001d002447*	
	*S2_220*	2	220613149		
	*S4_170*	4	170324384	*Zm00001d051812*	
	*S4_200*	4	200738088	*Zm00001d052781*	
	*S5_145*	5	145816043	*Zm00001d016131*	
	*S5_210*	5	210212211	*Zm00001d017927*	
	*S5_211*	5	211183684	*Zm00001d017928*	
	*S6_164a*	6	164808768	*Zm00001d038791, Zm00001d038806*	
	*S6_164b*	6	164811804	*Zm00001d038791, Zm00001d038806*	
	*S6_165*	6	165682422	*Zm00001d038843*	
	*S8_123*	8	123503579	*Zm00001d010672, Zm00001d010673*	
384 DH lines and 903 hybrids	*AX-90698604*	1	187,217,509		[137]
	*AX-108029030*	8	17,058,853		
	*AX-108089672*	10	3,276,832		
	*AX-107981937*	7	21,288,994		
	*AX-108109448*	8	167,766,262		
262 maize RILs	*AX-90974807*	5	S5_211766821		[138]
	*AX-91809638*	10	S10_13757681		
	*AX-91451802*	10	S10_145635671		
	*AX-91151225*	9	S9_138444899		
	*AX-90966401*	5	S5_179463254		
	*AX-91648757*	5	S5_37538103		
	*AX-90915192*	4	S4_227597917		
	*AX-90858848*	4	S4_13128859		
	*AX-91634643*	4	S4_208062434		
	*AX-90859313*	4	S4_14738387		
	*AX-90630900*	3	S3_178600050	*Zm00001d04270*	
	*AX-91411063*	3	S3_178254806	*Zm00001d04270*	
	*AX-91847440*	3	S3_38803403		
	*AX-91593556*	3	S3_218037573		
	*AX-90577822*	2	S2_13599625		
	*AX-91437579*	2	S2_11930489		
	*AX-90710167*	1	S1_233445080		
	*AX-90682835*	1	S1_124668841		
	*AX-91426201*	1	S1_174752484		
752 temperate maize genotypes	*Chr2:231,271,050*	2	231,271,050	*Zm00001d007424*	[32]
	*Chr4:78,851,667*	4	78,851,667	*Zm00001d050283* *Zm00001d050284* *Zm00001d050293*	
	*Chr4:173,863,109*	4	173,863,109	*Zm00001 d051914* *Zm00001d051893* *Zm00001d051869* *Zm00001d051884*	
	*Chr6: 169,030,253*	6	169,030,253	*Zm00001d039039* *Zm00001d039020* *Zm00001d039043* *Zm00001d039004*	

^a^ Chr. = chromosomes.

## Data Availability

No new data were created or analyzed in this study. Data sharing is not applicable to this article.
